# Sodium Selenite Accentuates the Therapeutic Effect of Adriamycin Prodrug (PADM) against Gastric Cancer

**DOI:** 10.1155/2019/2035682

**Published:** 2019-10-13

**Authors:** Shengquan Tan, Jiapeng Mo, Zixiong Zhang, Chuying Huang, Yi Zou, Jianhua Sun

**Affiliations:** ^1^Department of Gastrointestinal Surgery, The Central Hospital of Enshi Tujia and Miao Autonomous Prefecture, No. 158 Wuyang Avenue, Enshi, Hubei 445000, China; ^2^Hubei Selenium and Human Health Institute, The Central Hospital of Enshi Tujia and Miao Autonomous Prefecture, No. 158 Wuyang Avenue, Enshi, Hubei 445000, China; ^3^Department of Oncology, The Central Hospital of Enshi Tujia and Miao Autonomous Prefecture, No. 158 Wuyang Avenue, Enshi, Hubei 445000, China

## Abstract

Selenium has remained a controversial character in cancer research. While its antitumor effects have been widely demonstrated, further evidence is required to establish it as a robust treatment regime. Sodium selenite (SS), an inorganic selenium, reportedly affected the proliferation and redifferentiation of gastric cancer cells, but whether it could act as a complement to conventional chemotherapeutic drugs for combination therapy is uncertain. Herein, SGC-7901 and MGC-803 gastric cancer cells were treated with PADM (Ac-Phe-Lys-PABC-ADM), a prodrug of doxorubicin/adriamycin (ADM), and the combined antitumor effects of the two drugs were evaluated. Characterization after treatment revealed that although PADM exhibited antitumor effects individually by inhibiting the proliferation and migration of gastric cancer cells and inducing apoptosis, the addition of SS significantly amplified these effects. Furthermore, gastric cancer cell apoptosis triggered by the combined treatment of SS and PADM may involve the participation of mitochondrial apoptosis, as evidenced by the changes in mitochondrial morphology and occurrence of mitochondrial fission. Collectively, SS could be a strong complementary drug that accentuates the therapeutic potential of PADM in gastric cancer treatment and management, and its significance could contribute to unique and innovative anticancer strategies.

## 1. Introduction

With the rising incidence and high mortality of cancer, it has become the primary cause of death in China and emerged as a severe public health concern. Among various types of cancers, gastric cancer has the second highest rate of incidence and mortality in China. The total number of patients diagnosed with gastric cancer in China each year accounts for 42% of the worldwide number of cases, and the number of deaths exceeds two-thirds [1]. The strong heterogeneity of gastric cancer [2, 3] leads to a low rate of successful treatment, including surgical treatment, chemotherapy, radiotherapy, targeted therapy, and immunotherapy. Thus, optimizing the therapeutic scheme is an important step in the advancement of gastric cancer treatment.

Doxorubicin, or adriamycin (ADM), is one of the most important first-line drugs against cancer, with an effective rate of 40–50% when applied as a single-drug treatment regime. When ADM is combined with other chemotherapeutic drugs, it has an effective rate of up to 60–80% [4, 5]. Nevertheless, its clinical application is limited because its toxic effects increase with increasing dose [6, 7]. To address this issue, an effective and low-toxicity chemotherapeutic prodrug has been developed in the form of the ADM precursor Ac-Phe-Lys-PABC-ADM (PADM) [8]. In healthy tissues and peripheral blood, PADM is inactive, and it is only activated in the presence of excess cathepsin B, which is overexpressed on cancer cell membranes. Upon activation, PADM is cleaved to release free ADM molecules, which then exert their intended therapeutic impact [8]. In this way, the toxicity of ADM is mitigated in healthy tissues, ensuring that the drug only targets cancer cells and is inactive otherwise.

In addition to chemotherapeutic drugs, other compounds with proven antitumor properties have also been considered in the development of optimal anticancer strategies. Among them, selenium is an important component of selenoproteins and a necessary trace element in the human body. Under normal physiological conditions, relatively high selenium content (135 *μ*g/L) in the serum is positively related to the survival rate, whereas low selenium content (87 *μ*g/L) in the serum increases the risk of cancer and death [9]. Selenium is also involved in spermatogenesis [10–12] and antagonizes cardiovascular diseases [13, 14] and tumor function [15, 16]. Sodium selenite (SS), a common inorganic selenium, can induce superoxide anion production and lead to cancer cell apoptosis [17, 18]. SS has been highlighted as a promising alterative in cancer treatment, and its antitumor functions and therapeutic potentials warrant in-depth investigation.

In this study, the human gastric cancer cell lines SGC-7901 and MGC-803 were subjected to combination treatment of SS and PADM. Cell proliferation, apoptosis, and mitochondrial morphology were evaluated to determine the therapeutic effect of SS and PADM. In turn, the effect and mechanism of the drug combination on the apoptosis of SGC-7901 and MGC-803 cells were explored.

## 2. Materials and Methods

### 2.1. Cell Culture and Protocol

The SGC-7901 cell line was purchased from Bena Culture Collection (100674), MGC-803 cells were obtained from the Chinese Academy of Sciences, and GES-1 cells were purchased from Procell. After the cells were thawed, they were centrifuged at 800 rpm for 5 min to remove the supernatant and cultured in the RPMI-1640 medium (HyClone, Logan, UT, USA) containing 10% fetal bovine serum (FBS; Gibco; Thermo Fisher Scientific, MA, USA). Cells in the logarithmic growth were trypsinized with 0.25% trypsin (Bioswamp, Wuhan, China) in ethylenediaminetetraacetic acid and incubated in 6-well plates at 2 × 10^5^ cells per well. The cells were divided into three treatment groups: (1) 30 *μ*mol/L SS [19] for 24 h, (2) 3.6 *μ*g/L PADM [8] for 24 h, and (3) 30 *μ*mol/L SS for 24 h followed by 3.6 *μ*g/mL PADM for 24 h. Control SGC-7901 and MGC-803 cells were not treated with either SS or PADM. The morphology of the cells was visualized using a light microscope (TS-100F; Nikon, Tokyo, Japan).

### 2.2. Colony Formation Assay

A suspension of SGC-7901 or MGC-803 cells was prepared using 0.25% trypsin, and the cells were cultured in the RPMI-1640 medium containing 10% FBS. Then, the cells were diluted at a gradient of 50, 100, and 200 cells in 10 mL of the preheated medium (37°C) and shaken gently to ensure homogeneous distribution. The cells were cultured for three weeks at 37°C in an environment containing 5% CO_2_ at saturated humidity. When visible clones were observed in the culture dish, the supernatant was discarded and the cells were washed twice with phosphate-buffered saline (PBS). The cells were then fixed with 5 mL of 4% paraformaldehyde for 15 min, after which the fixative solution was removed and an appropriate amount of Giemsa solution was added to stain the cells for 10–30 min. The dye was rinsed with water, and the samples were dried. A light microscope (TS-100F; Nikon, Tokyo, Japan) was used to visualize the results.

### 2.3. Cell Scratch Assay

After SGC-7901 or MGC-803 cells were cultured in the serum-free medium in a 6-well plate at 1 × 10^5^ cells/mL for 24 h, SS and PADM were added to the cells for three days. Scratch lines were formed in the same direction using a 100 *μ*L pipette tip, and photos of the scratch were acquired under a microscope after 36 h of culture. The width of the scratch was determined using ImageJ.

### 2.4. Analysis of Cell Cycle and Apoptosis

Adherent SGC-7901 or MGC-803 cells were digested at 37°C in 0.25% trypsin (Bioswamp) and centrifuged at 1,000 ×*g* at 25°C for 5 min. The supernatant was discarded, and the cells were washed three times with PBS. The cells (1 × 10^5^ to 5 × 10^5^) were collected and resuspended in 200 *μ*L of the binding buffer (cat. no. C1052-1; Beyotime Institute of Biotechnology, Haimen, China) for analysis using the Cell Cycle and Apoptosis Analysis Kit (Beyotime) according to the manufacturer's protocol. Annexin V-FITC/propidium iodide staining was carried out for apoptosis, whereas propidium iodide analysis was applied for cell cycle progression. Analyses were performed using the LSR II Flow Cytometry System equipped with FACSDiva software 4.1 (BD Biosciences, Franklin Lakes, NJ, USA). Data were analyzed with ModFit LT software package version 4.0 (Verity Software House, Inc., Topsham, ME, USA).

### 2.5. Western Blot

SGC-7901 or MGC-803 cells were centrifuged at 1,000 ×*g* for 5 min at 37°C, washed three times with 1 mL of ice-cold PBS, and lysed using the Nuclear and Cytoplasmic Protein Extraction Kit (Beyotime) for 30 min at 4°C. The cell lysate was centrifuged at 15,000 ×*g* at 4°C for 10 min, and proteins were quantified using a bicinchoninic acid assay kit (Beyotime). A total of 15 *μ*g of proteins was separated by 10% sodium dodecyl sulfate-polyacrylamide gel electrophoresis, and the resolved proteins were transferred to a polyvinylidene fluoride membrane (EMD Millipore, Billerica, MA, USA). The membrane was blocked using 5% skim milk powder at 4°C overnight and incubated overnight at 4°C with primary antibodies against the following proteins: GAPDH (1 : 2,500; ab9485), cyclin-dependent kinase 4 (CDK4; 1 : 2,000; ab108357), Ki67 (1 : 5,000; ab92742), cyclin D1 (1 : 10,000; ab134175), cyclin E (1 : 1,000; ab33911), B-cell lymphoma 2 (Bcl-2; 1 : 1,000; ab32124), Bcl-2-associated X (Bax; 1 : 500; ab53154), cleaved caspase 3 (1 : 500; ab2302), cleaved caspase 9 (1 : 500; ab2324), p53 (1 : 1,000; ab131442), second mitochondria-derived activator of caspases (Smac; 1 : 1,000; ab8114), and apoptotic protease activating factor 1 (APAF1; 1 : 1,1000; ab8114) (all from Abcam, Cambridge, UK). After three washes in PBS containing 0.05% Tween 20 for 5 min each, the membrane was incubated with horseradish peroxidase-conjugated goat anti-rabbit immunoglobulin G H&L secondary antibody (1 : 2,000; ab6721; Abcam) at 4°C overnight. The results were quantified by Molecular Imager ChemiDoc XRS+ System 2.0 (Bio-Rad Laboratories, Hercules, CA, USA).

### 2.6. Transmission Electron Microscopy

A cell suspension was prepared using 0.25% trypsin, and the cells were cultured in the RPMI-1640 medium containing 10% FBS. Then, the cells were centrifuged at 500 ×*g* for 30 s at 37°C. After the supernatant was discarded, the cells were fixed for 4 h in 2.5% glutaraldehyde in PBS and rinsed three times for 5 min each using 0.1 M phosphoric acid. The fixed cells were dehydrated in ethanol at a concentration gradient (50%, 70%, and 90%) for 20 min at each concentration and then in a mixture of 90% ethanol and 90% acetone (1 : 1) for 20 min at 4°C and 90% acetone for 20 min at 4°C. The cells were sequentially embedded with a mixture of pure acetone and embedding liquid at 2 : 1 for 3 h at 25°C, in a mixture of pure acetone and embedding liquid at 1 : 2 overnight at 25°C, and in embedding liquid for 3 h at 37°C. The specimen was solidified at 37°C overnight, 45°C for 12 h, and then 60°C for 24 h. The prepared specimen was cut at a thickness of 60 nm using an ultramicrotome (EM UC7; Leica, Solms, Germany), stained with 3% uranyl acetate and lead citrate, and observed under a JEM-1200EX transmission electron microscope.

### 2.7. Statistical Analysis

Statistical analysis was conducted in SPSS 19.0 software (SPSS, Inc., Chicago, IL, USA) using the unpaired Student's *t*-tests. All experiments were performed in triplicate, and the data were expressed as mean ± standard deviation (SD). *P* < 0.05 was considered to be statistically significant.

## 3. Results

### 3.1. Inhibitory Effect of PADM on Gastric Cell Proliferation and Migration Was Amplified by SS

**Figure 1:**
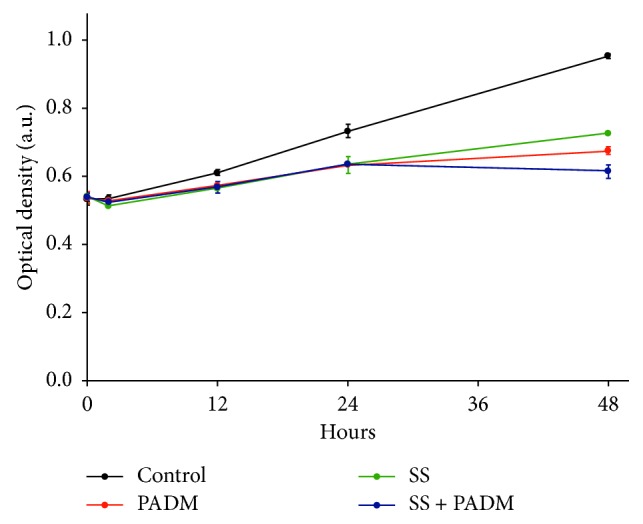
Effects of SS and PADM on normal gastric epithelial GES-1 cells. The CCK-8 assay was performed to determine the relative viability of GES-1 cells after treatment with SS and/or PADM for 2, 12, 24, and 48 h. All data are presented as mean ± SD (*n* = 3).

The effect of SS and PADM on normal cell survival and proliferation was first determined in a gastric epithelial cell line GES-1 using the CCK-8 assay (Figure 1). GES-1 cells continued to proliferate for up to 48 h even after SS and/or PADM treatment, though at a slower rate than control cells. It is reasonable that the therapeutic agents have a certain degree of effect on normal cells, but they did not suppress normal cell proliferation. This indicates that SS and PADM are safe and nontoxic to normal cells, setting the foundation for our subsequent experiments using gastric cancer cell lines.

**Figure 2:**
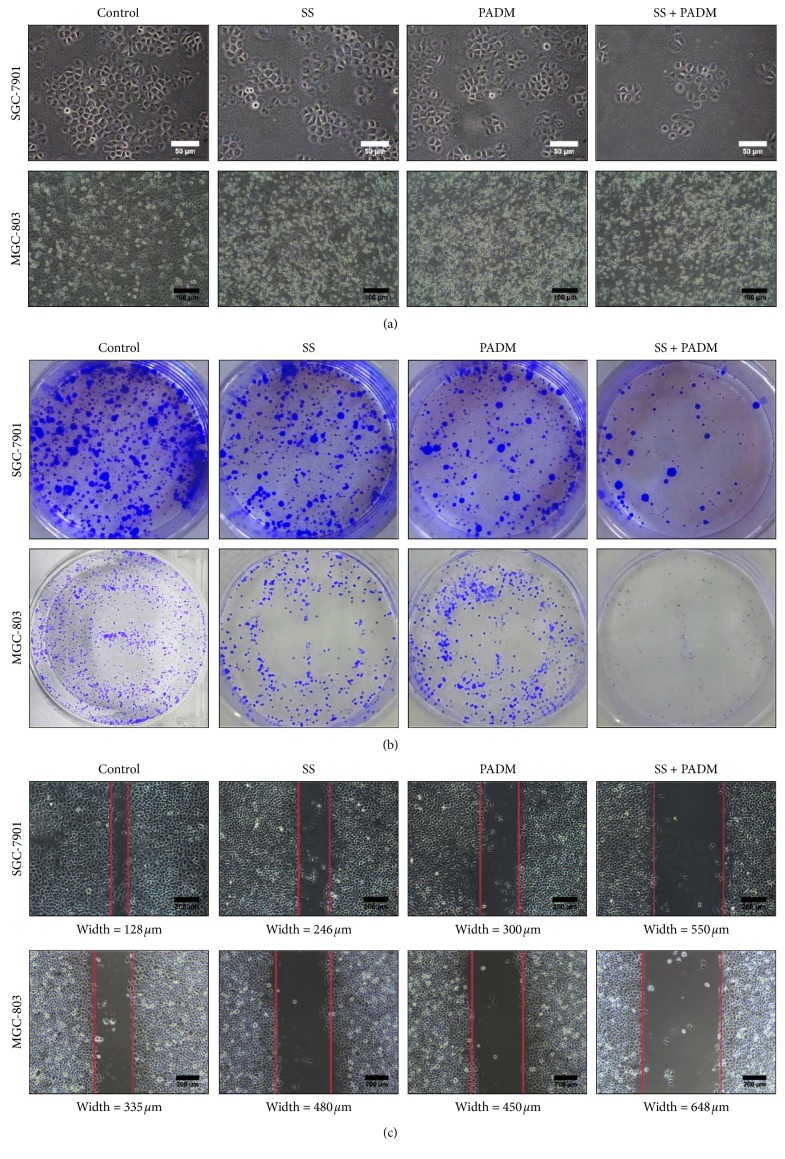
Evaluation of the proliferation and migration of SGC-7901 and MGC-803 gastric cancer cells. (a) The proliferation of SGC-7901 and MGC-803 gastric cancer cells was observed using light microscopy. The number of SGC-7901 cells showed a decrease with SS and/or PADM treatment, and the number of dead MGC-803 cells (round, hollow cells) was increased with SS and/or PADM treatment. Scale bar = 50 *μ*m for SGC-7901 cells and 100 *μ*m for MGC-803 cells. (b) The proliferation of SGC-7901 and MGC-803 cells was detected by the colony formation assay (magnification 40x). Both SGC-7901 and MGC-803 cells showed a clear decrease in colony formation with SS or PADM treatment, and the combination of SS and PADM accentuated the effects of the individual components. (c) The migration of SGC-7901 and MGC-803 cells was determined by the scratch assay. Gap closure efficiency was reduced by SS or PADM in both cell lines, and the combination of SS and PADM accentuated the effects of the individual components. Scale bar = 200 *μ*m for both cell lines.

The proliferative potential of SGC-7901 and MGC-803 gastric cancer cells was then detected by the colony formation assay. Individual treatment of PADM decreased cell survival and proliferation (Figure 2(a)) and colony formation (Figure 2(b)), and this effect was accentuated with the combined treatment of SS and PADM. Furthermore, the migration of SGC-7901 and MGC-803 cells was reduced after treatment with SS or PADM, as demonstrated by the lower degree of gap closure in the scratched cell layer compared to that formed by nontreated cells (Figure 2(c)). Correspondingly, the combined antimigration effect of SS and PADM was greater than that of the individual components in both cell lines, suggesting that SS amplified the inhibitory potential of PADM in gastric cancer cell growth.

### 3.2. SS Promoted G1 Phase Blocking in Gastric Cancer Cells in conjunction with PADM

**Figure 3:**
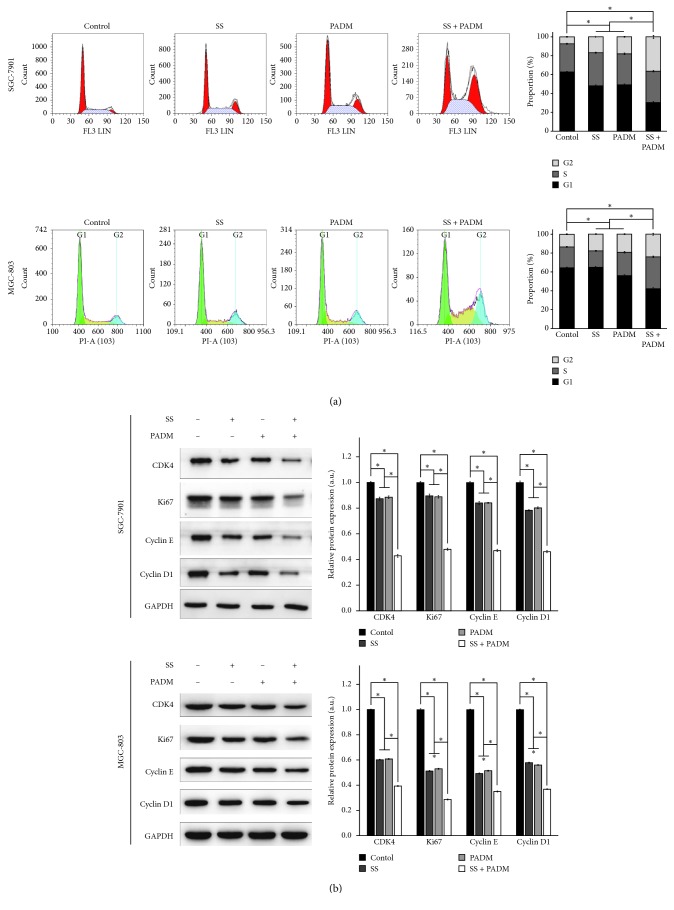
G1 phase blocking and cell cycle progression in SGC-7901 and MGC-803 cells. (a) Cell cycle progression in SGC-7901 and MGC-803 cells was detected by flow cytometry. G1 phase blocking was observed in both cell lines when treated with SS or PADM, and the combined effect of SS and PADM was greater than that of the individual components. (b) Proteins associated with cell cycle (CDK4, Ki67, cyclin E, and cyclin D1) were examined by western blot. Decreased expression of these proteins was observed in both cell lines when treated with SS or PADM, and the combined effect of SS and PADM was greater than that of the individual components. All data are presented as mean ± SD (*n* = 3). ^*∗*^*P* < 0.05.

The cell cycle progression of SGC-7901 and MGC-803 cells was evaluated by flow cytometry. Individual treatment of SS or PADM significantly reduced the percentage of cells in the G1 phase (*P* < 0.05), whereas that in the G2 phase was elevated compared with the control cells (*P* < 0.05). Effectively, combined treatment of SS and PADM exerted a greater impact on cell cycle progression than did the individual components (*P* < 0.05) (Figure 3(a)). This finding suggested that SS markedly enhanced the effect of PADM in inducing G1 phase blocking, in turn promoting cell cycle arrest at the G2/M phase. Western blot revealed that the expression of the cell cycle-associated proteins CDK4, Ki67, cyclin E, and cyclin D1 was decreased after individual and combined treatment of SS and PADM (Figure 3(b)).

### 3.3. SS Accentuated Apoptosis and Mitochondrial Fission in PADM-Treated Gastric Cancer Cells

**Figure 4:**
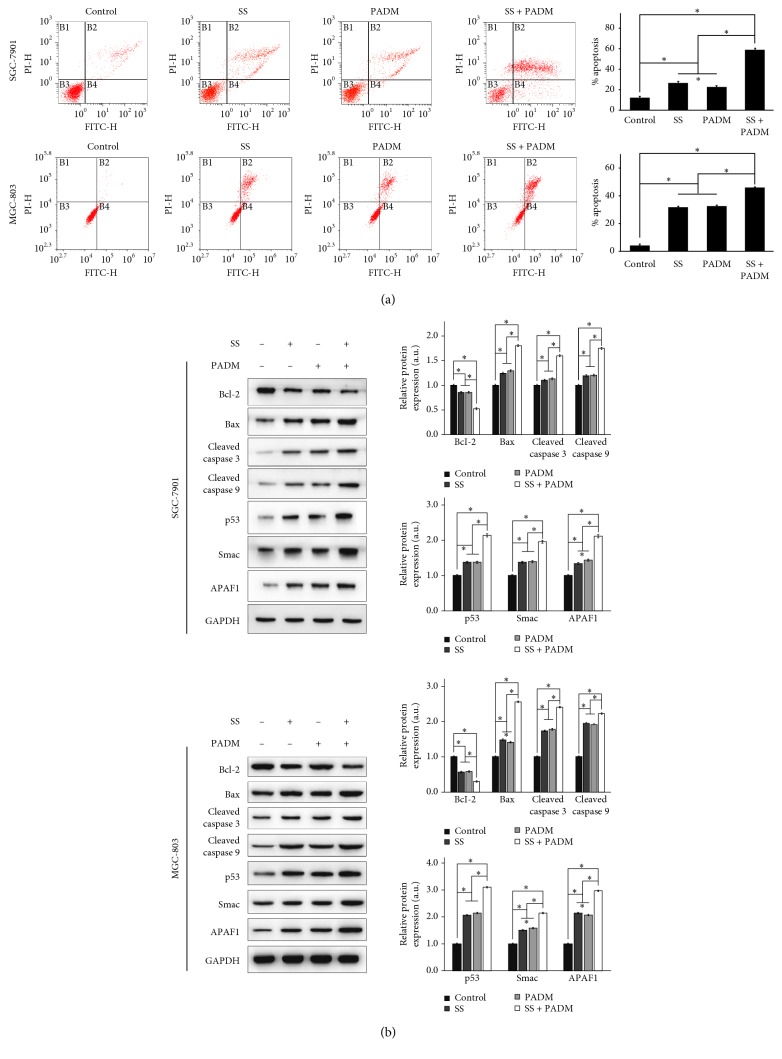
Apoptosis of SGC-7901 and MGC-803 cells. (a) The percentage of SGC-7901 and MGC-803 cell apoptosis was detected by flow cytometry. The percentage of apoptotic cells was increased in both cell lines when treated with SS or PADM, and the combined effect of SS and PADM was greater than that of the individual components. (b) Proteins associated with apoptosis (Bcl-2, Bax, cleaved caspase 3, and cleaved caspase 9) and mitochondrial apoptosis (p53, Smac, and APAF1) were examined by western blot. Downregulation of Bcl-2 and upregulation of Bax, cleaved caspase 3, cleaved caspase 9, p53, Smac, and APAF1 were observed in both cell lines after treatment with SS or PADM, and the combined effect of SS and PADM was greater than that of the individual components. All data are presented as mean ± SD (*n* = 3). ^*∗*^*P* < 0.05.

In accordance with the results of cell cycle progression, flow cytometry was performed to assess gastric cancer cell apoptosis (Figure 4(a)). Compared with nontreated control cells, the percentage of SGC-7901 and MGC-803 cell apoptosis was increased by individual administration of SS and PADM (*P* > 0.01), and their combined effect was amplified compared to that of individual treatment (*P* < 0.001). In turn, proteins associated with cell apoptosis (Bcl-2, Bax, cleaved caspase 3, and cleaved caspase 9) and mitochondrial apoptosis (p53, Smac, and APAF1) were examined by western blot. The expression of the antiapoptosis protein Bcl-2 was downregulated, whereas the expression levels of the proapoptotic Bax, cleaved caspase 3, and cleaved caspase 9 were upregulated by combined SS and PADM treatment. Similarly, p53, Smac, and APAF1 were significantly upregulated by combination treatment compared with individual treatment (Figure 4(b)).

**Figure 5:**
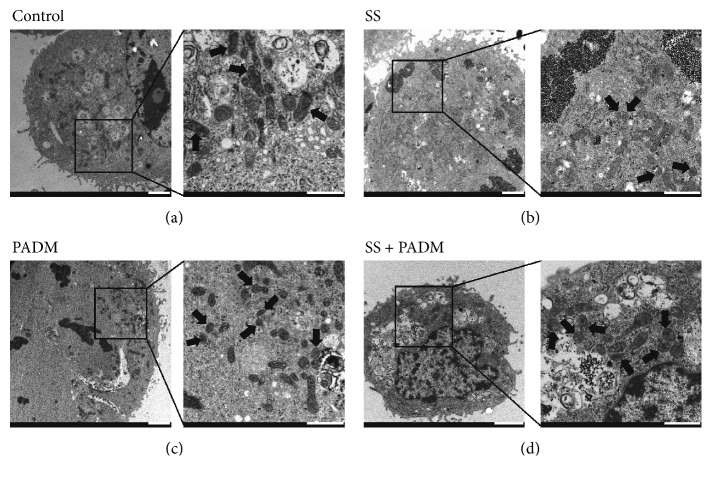
Morphology of subcellular mitochondria in SGC-7901 cells. The morphology and structure of mitochondria were observed by transmission electron microscopy. Dark, oval, or round features in the images represent mitochondria. In each group, a magnified image of the rectangular area is shown. Black arrows in the magnified images represent subcellular mitochondria. Scale bar = 2 *μ*m and 1 *μ*m (for magnification).

To examine the role of mitochondrial function more closely, the mitochondria in treated SGC-7901 cells were observed by transmission electron microscopy (Figure 5). The subcellular mitochondrial morphology was normal in the control cells, wherein mitochondria were present in an oval, elongated shape. However, mitochondrial fission appeared to occur when the cells were treated with PADM or SS. This was demonstrated by the individual round mitochondria that are close to each other, which could be indicative of the fission process and implicate mitochondrial apoptosis. When SS was administered in conjunction with PADM, mitochondrial fission became more obvious than in the cases of individual treatment. Collectively, these data suggested that the addition of SS accentuated the effect of PADM in causing apoptosis in SGC-7901 cells, and the mitochondrial apoptosis pathway may be involved in this process.

## 4. Discussion

PADM has recently emerged as an alternative to ADM, offering a low-toxicity strategy in the treatment of gastric cancer. In the PADM molecule, the specific substrate Ac-Phe-Lys is linked to ADM via PABC (para-aminobenzyloxycarbonyl), which acts as a spacer. Upon exposure to cathepsin B, which is present on cancer cell membranes, the Lys-PABC bond is cleaved, and subsequent self-hydrolysis of PABC allows free ADM to be released [8]. Cathepsin B is an important class of enzymes related to tumor progression and is a candidate target for molecular therapy. The related characteristics of cathepsin B have been applied in the development of new anticancer drugs based on molecular targeting [20]. The use of PADM takes advantage of the biological characteristics of local cathepsin B release in the process of gastric cancer invasion and metastasis [21], demonstrating that the toxicity of PADM was significantly reduced under exposure to low levels of cathepsin B. In turn, safe doses of PADM prevented the peritoneal metastasis of gastric cancer in nude mice, reduced toxicity to the heart, liver, and kidney, and suppressed the overall toxic and side effects [8].

Selenium is a strong antioxidant with proven anticancer properties, but several selenoproteins, such as thioredoxin reductase 1 and selenoprotein 15, have been shown to exhibit dual tumor-promoting and tumor-suppressive functions [22]. In cancer research, selenium has provoked controversy as a treatment scheme, and opinions have been split as to whether the use of selenium is beneficial in cancer prevention. Some studies have reported on the prominent link between selenium deficiency and cancer incidence [23, 24], but null association has been reported in others [25, 26]. Regional variations may also need to be taken into consideration when addressing this issue. Taken together, there is a lack of evidence drawing a robust link between selenium and cancer. With the emergence of combination therapy as a highlight in tumor treatment and management, various compounds have been suggested as complements to conventional chemotherapeutic drugs, either to maximize the effects or to minimize the toxicity of these drugs. Among these compounds, SS has gained attention as a promising candidate, and its safety and efficacy have been evaluated in clinical pharmacokinetic studies [27]. At a dose lower than 2 *μ*M, SS promoted cell survival, while a hypertrophic dose of selenium (higher than 5 *μ*M) induced apoptosis in a variety of tumor cells [28]. Selenite can also selectively eliminate tumor cells and have few side effects on normal cells. The intravenous injection of selenite containing 1.5–4.0 *μ*C/kg ^75^Se into patients harboring various types of tumors showed that selenite was selectively enriched in patients with intracranial and thoracic and abdominal tumors [29]. In addition, studies have shown that SS is effective in killing cancer cells [30] and is closely related to the production of reactive oxygen species [31].

This study focuses on the important role of SS in accentuating the therapeutic potential of PADM, aiming to achieve the goal of minimal toxicity and maximized efficacy against tumor cells. While PADM alone was able to promote gastric cancer cell apoptosis, SS acts as a complementary agent to further emphasize the antitumor effects. The combined treatment strategy has advantages that contribute to the development of anticancer therapeutics. In particular, the synergistic effects of SS and PADM are stronger than those of the individual components, and in the meantime, toxicity induced by conventional drugs such as ADM could be mitigated with the use of prodrugs, PADM in this case. The findings of this study also elucidate that the apoptotic mechanisms were due to an increase in mitochondrial fragmentation through the process of fission, which is a required step in apoptosis [32]. Moreover, the activation of caspases 3 and 9 has been associated with mitochondrial remodeling and apoptosis [33]. The upregulation of cleaved caspases 3 and 9 by SS and PADM not only serves as a sign of cell apoptosis but also supports the hypothesis that the mitochondrial apoptosis pathway is a vital component of the antitumor effects of the proposed combination therapy.

## 5. Conclusions

The findings of this study presented a novel strategy of combination therapy (SS and PADM), which inhibited the proliferation and promoted the apoptosis of gastric cancer cells. Importantly, the prominent role of SS in accentuating the therapeutic effects of PADM was demonstrated from various perspectives and implicates the involvement of mitochondrial apoptotic pathways through the fission process. The specific mechanisms and signaling pathways involved in the combination treatment of SS and PADM *in vitro* require additional exploration.

## Data Availability

The data used to support the findings of this study are available from the corresponding author upon request.
